# Associations between Celiac Disease, Extra-Gastrointestinal Manifestations, and Gluten-Free Diet: A Narrative Overview

**DOI:** 10.3390/nu16121814

**Published:** 2024-06-09

**Authors:** Antonella Santonicola, Herbert Wieser, Carolina Gizzi, Carlo Soldaini, Carolina Ciacci

**Affiliations:** 1 Gastrointestinal Unit, Department of Medicine, Surgery and Dentistry “Scuola Medica Salernitana”, University of Salerno, 84131 Salerno, Italy; asantonicola@unisa.it (A.S.); carolinagizzi@libero.it (C.G.); carlo.soldaini@outlook.com (C.S.); 2 Hamburg School of Food Science, Institute of Food Chemistry, University of Hamburg, 20146 Hamburg, Germany; h.wieser2@gmx.de

**Keywords:** celiac disease, extraintestinal manifestations, gluten-free diet, cardiovascular, otorhinolaryngeal, skin, muscles and joints, bones, adults, children

## Abstract

Millions of children and adults worldwide suffer from undiagnosed and untreated celiac disease (CeD). The clinical picture of CeD is highly heterogeneous and comprises manifestations that can affect almost the whole body. This narrative overview is aimed at characterizing diseases and complaints that are associated with unrecognized CeD and that frequently involve sites other than the gastrointestinal (G.I.) tract, i.e., dental, otorhinolaryngological, and ocular complications; skin and hair abnormalities; afflictions of the bones, joints, and muscles; cardiovascular affectations; kidney diseases; neuro-psychiatric disorders; and gynecological–obstetrical manifestations. The association between CeD and extra-GI manifestations is frequently overlooked, which leads to a delay in diagnosis. Most CeD-mediated disorders can be treated with a strict gluten-free diet (GFD), but some of them are irreversible unless CeD is diagnosed in time. Some manifestations can be classified as risk factors for CeD, and CeD screening tests for affected patients should be selectively considered. Apart from gastroenterologists, specialists in other medical disciplines can play an important role in identifying people with unrecognized CeD and may help prevent its progress and long-term complications. Further comprehensive investigations are necessary to clarify the pathogenesis of extra-GI manifestations and the effect of a GFD.

## 1. Introduction

Celiac disease (CeD) is a chronic immune-mediated enteropathy that occurs in genetically predisposed individuals when exposed to a protein, gluten, in wheat, rye, barley, and oats [1]. Both human leukocyte antigen (HLA) class II alleles (HLA-DQ2.5, -DQ2.2, and -DQ8) and non-HLA genes contribute to its genetic predisposition. Several “second hits,” such as infections, imbalanced intestinal microbiota, and increased intestinal permeability, have been linked with the onset of CeD. The prevalence of CeD in the general population is about 1.7% based on positive serology, and 0.7% based on biopsy-confirmed diagnosis [2]. Only black people from sub-Saharan Africa seem to be hardly affected. Both children and adults can be affected by CeD at any age, and there is a higher prevalence in women than in men (ratio 2:1–3:1). First-degree relatives and individuals with autoimmune and genetic disorders have a higher risk of CeD. The defective digestion of gluten in the gastrointestinal (G.I.) tract resulting in the generation of immunogenic peptides, the transcellular and paracellular movement of gluten peptides through the small bowel epithelium, and the activation of both adaptive and innate immune responses to gluten peptides in the lamina propria are among the pathogenetic mechanisms of CeD [3]. The typical pathological features of the duodenal mucosa in CeD are villous atrophy (“flattened mucosa”), crypt hyperplasia, and increased intraepithelial lymphocytes. Strict lifelong compliance to a gluten-free diet (GFD) is currently the only available therapy for CeD.

The diagnosis of CeD requires a high level of clinical knowledge and a multi-step procedure consisting of accurate anamnesis, serology, small intestinal histology, response to a GFD, and optionally genetic status [4]. Due to its diagnostic complexity, CeD is one of the most under-diagnosed disorders worldwide. As reported by a systematic review and meta-analysis evaluating 291,969 study participants, the pooled prevalence of undiagnosed CeD is 0.42% in men and 0.59% in women [5]. Therefore, there are around 22 million women and 16 million men worldwide with undetected and thus untreated CeD. Moreover, delays in diagnosis are unfortunately common. A comparison of questionnaires designed in 2006 and 2015 and responded to by 1600 diagnosed patients with CeD from the U.K. showed that the period from first symptoms to diagnosis had not significantly changed (13.4 vs. 12.8 years) [6].

Although CeD was initially considered to be a children’s disease characterized by chronic diarrhea and malabsorption of nutrients, it was afterward identified as a chronic disorder also affecting adults at any age. There is a wide spectrum of clinical manifestations of CeD due to its multi-system involvement [7]. Several factors, such as the location, the extension, and the degree of mucosal damage, might influence the severity of symptoms. The manifestations associated with CeD can be roughly divided into gastrointestinal and extra-gastrointestinal. In some cases, only extra-GI manifestations are present, significantly delaying diagnosis. In principle, any organ, from the teeth, skin, and bones to the reproductive and neuropsychiatric systems, can be affected by extra-GI manifestations.

This manuscript aims to give an overview of the numerous extra-GI manifestations associated with untreated CeD. Medical specialists outside of gastroenterology should point to the possible association between CeD and extra-GI disorders, and thus they might help to identify people with unrecognized and untreated CeD combined with the prevention of its progress and complications in the long term.

## 2. Materials and Methods

**Figure 1:**
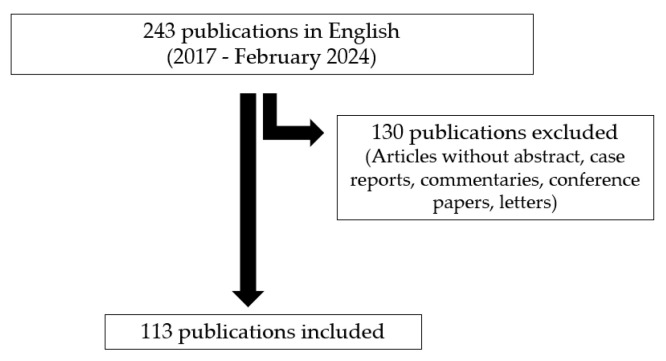
PRISMA flow diagram of the search procedure.

PubMed database searches were conducted for articles published in English from 2017 to February 2024 using the keywords “c(o)eliac disease” in combination with “reviews” and “manifestations”. A total of 243 publications were retrieved, and 44 of them were selected. An additional 69 papers, published from 2008 to 2023, were identified by cross-referencing from the retrieved reviews and personal files on CeD (Figure 1). Articles without abstracts, such as commentaries, case reports, letters, and conference papers, were excluded. Around 85% of the cited 113 articles were published between 2017 and 2023.

## 3. Oral Cavity, Nose, and Ears

**Figure 2:**
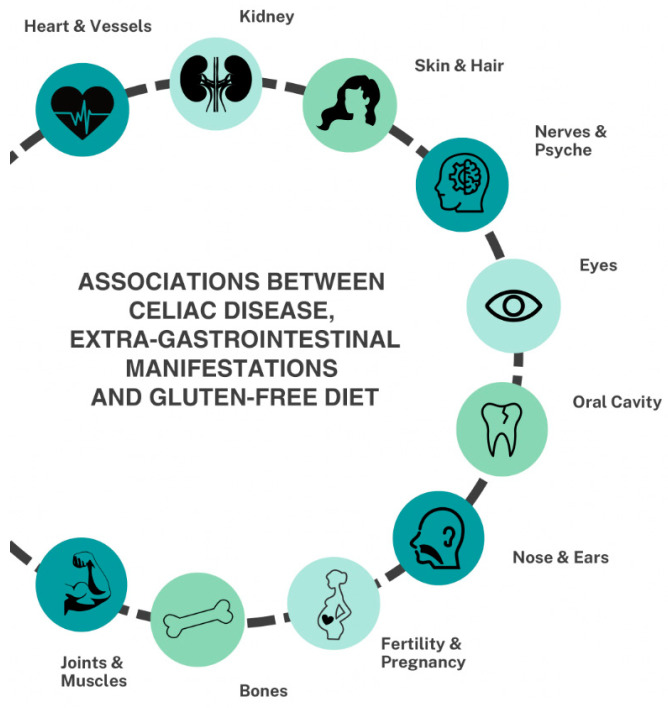
The extra-GI manifestations associated with untreated CeD.

Several dental manifestations have been described in CeD in particular, such as delayed dental eruption and maturity in children, dental enamel defects (DEDs), dental caries, dental plaque, and periodontitis in children and adults [8] (Figure 2; Table 1). A case–control study from Saudi Arabia evaluated the presence of DEDs in 104 children with CeD and 104 non-CeD children [9]. The results demonstrated that more children with CeD (70.2%) had DEDs than controls (34.6%). Therefore, the diagnosis of CeD should be suspected by pediatric dentists in young patients with DEDs. To examine dental manifestations in adult Indian CeD patients in comparison to healthy individuals, 118 patients with biopsy-proven CeD (group A: 36 patients at diagnosis; group B: 82 patients after a GFD for at least one year), and 40 controls (group C) were recruited for dental surgery [10]. All in all, 66.9% of group A and 69.4% of group B had DEDs compared to 20% of group C. The similar prevalence of DEDs in CeD patients with new diagnoses and in those on a longtime GFD demonstrated that DEDs have already been established in adult patients and appear to be irreversible. The malabsorption of micronutrients, especially calcium and vitamin D, has been proposed as the main cause of DEDs. Early diagnosis of CeD and treatment with a GFD might prevent the development of DEDs.

A couple of studies have demonstrated that CeD children more frequently show delayed dental eruption and maturation than healthy children. Early diagnostics for CeD and a strict GFD may help prevent these complaints. Recent investigations supplemented older contradictory studies on the association between CeD and dental caries, dental plaque, and periodontitis; however, clear conclusions could not be reached [8]. All in all, the link between CeD and DED has been proved. Patients with DEDs, even in the absence of G.I. complaints, should be investigated for CeD, and dentists could play a fundamental role in recognizing undiagnosed CeD.

Otorhinolaryngological (ORL) manifestations in CeD, occurring in both children and adults, are relatively rare, and their association with CeD is mostly overlooked by physicians [11]. It is well known that several disorders, such as recurrent aphthous stomatitis (RAS), dry mouth, atrophic glossitis, angular cheilitis, and burning tongue, affect the soft tissue of the oral cavity in active CeD [12,13]. Multiple studies have demonstrated that RAS is highly frequent both in children and adults with CeD. In a group of 740 Dutch adults with CeD, for instance, RAS was more frequently reported compared to a comparison group of 270 healthy subjects (35% vs. 23%) [14]. A Turkish study on 62 CeD children and 64 controls revealed a prevalence of RAS of 31% in CeD children, while none of the controls had RAS [15]. Vice versa, several investigations demonstrated a higher prevalence of CeD in subjects suffering from RAS compared to controls. Yilmaz et al. evaluated the prevalence of CeD in Turkish children with RAS [16]. Among 108 pediatric patients, 3 (2.7%) were diagnosed with CeD, which was nearly six times more frequent compared to healthy children within the Turkish population (0.5%). The effect of a GFD on RAS development is still in discussion and should be clarified in future investigations. To conclude, the presence of enamel hypoplasia and RAS may suggest the presence of CeD (“risk indicators”) [17].

Several investigations found that atrophic glossitis, geographic tongue, angular cheilitis, aphthous ulcers, and xerostomia are more frequent in CeD. A Greek study of 45 children and adolescents with CeD and 45 matched healthy controls showed a significant difference in the prevalence of aphthous ulcers in the group of CeD patients (40%) than in healthy controls (4.4%) [18]. In the same cohorts, three CeD patients (6.7%) and none of the control group suffered from geographic tongue. A study from Portugal, including 80 children and adolescents with CeD and 80 matched healthy subjects, revealed that the prevalence of atrophic glossitis in the CeD cohort was 6.3%, while none of the controls had this complaint [19]. Similar results were obtained for the prevalence of angular cheilitis. Liu et al. investigated 20 adult Danish CeD patients and 20 matched healthy controls to determine if the salivary glands are involved in CeD [20]. Xerostomia, mucosal lesions, focal lymphocytic sialadenitis, and dry/cracked lips occurred more frequently and extensively in the CeD patients compared to the healthy controls. The evaluation of 65 Iranian children with CeD and 60 matched healthy controls showed that the frequency of xerostomia was significantly higher in the group with CeD (15%) than the controls (5%) [21]. Reports on the effect of CeD on salivary properties such as flow rate, pH, and buffering capacities are contradictory, and further investigations are necessary.

A literature review including 17 studies was conducted by Karunaratne and Karunaratne to identify the relationships between CeD and ear and nose manifestations [22]. Research targets included sensorineural hearing loss, obstructive sleep apnea, nasal septal perforation, and epistaxis. The main limitations in this area were the partly small number of studies and the contradictory results. The majority of research has concerned sensorineural hearing loss. A systematic narrative review on the association between CeD and sensorineural hearing loss, including 10 studies of children and adults, was published by De Luca et al. [23]. Some examinations demonstrated a significant effect of CeD on the auditory system, while others did not.

In conclusion, several ORL manifestations are more common in CeD patients compared to healthy individuals. A GFD may result in sufficient symptom resolution for most manifestations; only sensorineural hearing loss appears to be progressive and permanent. The pathophysiological link between ORL disorders and the effect of a GFD should be further investigated.

## 4. Eyes

CeD has been associated with a number of ocular complaints and disorders in both adults and children [24], including dry eye, nyctalopia, thyroid-associated orbitopathy, cataracts, central retinal vein occlusion, uveitis, and neuro-ophthalmic manifestations (Figure 2; Table 1). Immunogenic factors, the cross-reactivity of cell antigenic epitopes, and vitamin deficiencies, among others, have been discussed as mechanisms for ocular involvement in CeD. The following recent investigations exemplarily demonstrate the close relation between CeD and several defects in ocular structures. An ocular imaging-based study of 36 Turkish adults with CeD and 35 healthy controls, using a complete ophthalmologic examination, was performed by Dömez Gün et al. [25]. The results demonstrated that endothelial cell density, central macular thickness, anterior chamber depth, and anterior chamber volume were significantly lower in CeD patients. An Italian study by De Bernardo et al. evaluated the differences in choroidal structure between 74 CeD adult patients and 67 healthy subjects [26], and demonstrated significant differences for the total subfoveal area, stromal subfoveal area, luminal subfoveal area, and subfoveal choroidal thickness, but not for choroidal vascularity index.

Two Turkish cohorts of 31 children with classical CeD and 34 matched control children were examined by the group of Karatepe Hashas [27]. They showed a significantly lower anterior chamber depth, a reduced anterior chamber volume, lower Schirmer value and decreased break-up time test, and reduced retinal nerve fiber layers in the eyes of the children with CeD compared to the controls. A total of 43 Turkish children with CeD and 48 healthy controls underwent comprehensive eye examinations using enhanced depth imaging optical coherence tomography [28]. The results revealed that all layers of subfoveal, nasal, and temporal choroid were significantly thinner in the CeD group than in the control group. There were not any significant differences between the CeD patients and controls for ganglion cell complex thicknesses.

In summary, there is a relationship between CeD and ocular manifestations. Unfortunately, pathogenesis and the role of a GFD in the development or prevention of these manifestations are not definitively clarified and need further investigation.

## 5. Skin and Hair

Over recent decades, multiple investigations indicated that untreated CeD could affect skin and hair (Figure 2; Table 1). Research on skin disorders such as dermatitis herpetiformis, chronic urticaria, atopic dermatitis, psoriasis, and rosacea has been by far predominant. Dermatitis herpetiformis (D.H.) or Duhring disease is also defined as “skin CeD”, since it is the cutaneous counterpart of CeD [29]. D.H. is characterized by severe cutaneous itching and burning due to the herpetiform clusters of papules and vesicles on various parts of the body. The prevalence of D.H. is much lower than that of CeD, and in contrast to CeD, DH appears more common in males than in females and is rare in children. A valid hypothesis for the immunopathogenesis of D.H. is that it starts from latent or manifest CeD in the small intestine and evolves into an immune complex deposition in the papillary dermis [30]. Patients with D.H. diagnosis should start a lifelong GFD, although symptoms may respond slowly.

Besides the well-known association of CeD with D.H., there have been numerous reports on other skin affectations related to CeD that may improve with a GFD [31]. However, the results were at times inconsistent regarding the possibly increased frequency of skin complaints in CeD patients compared to non-CeD subjects. Lebwohl et al. demonstrated an increased risk for acne, eczema, psoriasis, vitiligo, alopecia areata, and urticaria in CeD compared to controls [32]. In contrast, a Finnish study, including 327 CeD patients and 382 non-CeD controls, demonstrated that CeD patients were at no increased risk of atopic dermatitis, acne, rosacea, psoriasis, alopecia areata, vitiligo, or chronic urticaria [33].

Over the last years, several studies have linked atopic dermatitis (A.D.) with CeD. The symptoms of A.D. include cutaneous itch and pain, sleep disturbance, fatigue, and mental health symptoms. Ress et al. analyzed the prevalence of CeD in 351 children with A.D. in comparison to the general pediatric population and showed a four-fold greater risk of developing CeD in patients with A.D [34]. Shalom et al. evaluated the potential association between A.D. and CeD in a broad community-based population in Israel and demonstrated that A.D. was associated with a significantly higher prevalence of CeD [35].

Psoriasis is a chronic skin disease characterized by cutaneous lesions and less commonly pustulation, potentially involving all the body. In contrast to some previous investigations, recent studies revealed the relationship between CeD and psoriasis to be unequivocally present. Patients with psoriasis showed an approximately three-fold increased risk of CeD compared to controls [36]. A subsequent systematic review confirmed the significant association between psoriasis and CeD [37]. Therefore, patients with psoriasis should be screened for CeD.

Rosacea is an inflammatory skin condition characterized primarily by persistent or recurrent episodes of centrofacial erythema, with women being more affected than men. Some recent studies have suggested a link between rosacea and CeD but data are limited. In a nationwide Danish study, 49,475 patients with rosacea and 4,312,213 general population controls were identified using nationwide administrative registers in order to investigate the association between rosacea and different G.I. disorders [38]. Regarding CeD, its prevalence was significantly higher among patients with rosacea when compared to controls.

Although several studies have suggested a relationship between CeD and vitiligo, their conclusions are still conflicting. Vitiligo is a chronic autoimmune disorder that causes patches of skin to lose pigment or color, which tends to occur on the extremities. Zhang et al. summarized the literature on this relationship, supporting the association between CeD and vitiligo, and the potential benefit of a GFD for patients affected by vitiligo [39].

The association of alopecia areata (A.A.) with CeD and the possible advantages of a GFD are well documented [40]. The hair of A.A. patients can be thin and slow-growing; its color might be influenced by the bowel malabsorption of pantothenic acid or manganese. A.A. patients have patches of hair loss and also often fragile, irregular nails that break easily. Nails grow slowly and can have color changes and horizontal ridges. Nail symptoms are partially or fully caused by nutrient deficiencies, including vitamins, minerals, essential fatty acids, and amino acids. Since the 1990s, there have been a couple of reports on the association between A.A. and CeD. Although the estimated prevalence rate of CeD in patients with A.A. is similar to that found in the general population, the fact that A.A. improves and even disappears with a GFD may indicate the possible existence of a relationship with an undiagnosed CeD [31].

In summary, D.H. is well known as an effect of CeD on the skin and can be cured with a GFD. More recently, CeD has been related to the development of A.D., psoriasis, rosacea, and A.A. Their pathogenesis and the effect of a GFD should be further investigated.

## 6. Bones

Bone manifestations such as decreased bone density, bone pain and fracture, osteopenia, and osteoporosis are common in CeD patients (Figure 2; Table 1). The underlying mechanisms remain incompletely understood, although several mechanisms, such as the malabsorption of calcium and vitamin D and the secretion of pro-inflammatory cytokines, have been suggested [41]. Decreased bone mineral density (BMD) has been frequently diagnosed in both children and adults with active CeD. A systematic review and meta-analysis, based on 12 studies published between 1996 and 2017, indicated that the bone mineral content and areal BMD of children and adolescents with CeD were significantly lower as compared to healthy controls [42]. Another systematic literature review showed that young adults between 20 and 35 years of age had an average lower BMD at CeD diagnosis compared to healthy controls [43]. GFD adherence usually results in the partial recovery of bone density by one year and full recovery by the fifth year associated with duodenal mucosa healing. However, in older CeD patients and in those with late-onset disease, the recovery of BMD is not guaranteed despite a GFD [44]. Some investigations have shown an increased risk of fractures in adult CeD patients [45]. For example, Hjelle et al. evaluated the association between CeD and fractures by analyzing blood from 400 Norwegian patients with distal fractures, aged 40 years or above, and 197 controls who had never suffered a fracture, for the level of IgA transglutaminase antibodies (TGAs) typical of active CeD [46]. The results demonstrated that 2.5% of the fracture patients had positive IgA TGAs compared to 1% in the control group.

Numerous studies demonstrated that osteopenia and osteoporosis are common comorbidities in adults with CeD. A systematic review of the literature, for instance, including 563 men and premenopausal women with CeD, examined in different countries, showed that 14.4% suffered from osteoporosis and 39.6% from osteopenia [47]. The examination of 214 Italian adult patients newly diagnosed with CeD revealed that 42% of patients developed osteopenia and 18% osteoporosis [48]. Male gender, age ≥45 years, underweight, and Marsh 3C intestinal histology were significantly associated with osteoporosis. In a group of 250 patients from the Netherlands, at CeD diagnosis, osteoporosis and osteopenia were found in 23% and 35%, respectively [49]. Skoracka et al. proposed that genetic, immunological, dietary, and environmental factors as well as unfavorable gut microbiota may be responsible for the development of osteoporosis [50]. One study reported that Caucasian adults with symptoms/signs of undiagnosed CeD in the early years of life showed foreheads larger than those of CeD patients with an onset of CeD later in life [51]. This alteration is a clinical sign that should be included among the extra-GI manifestations of CeD, as it is present with a frequency comparable to short stature and is a better predictor of CeD than other signs such as RAS, recurrent abortion, and dental enamel hypoplasia.

In summary, untreated CeD is frequently associated with reduced BMD in children and adults, and with fracture, osteopenia, and osteoporosis in adults. The early diagnosis of CeD and treatment with a strict GFD may either protect patients from bone diseases or may resolve them when they are already present. However, in older CeD patients and those with late CeD onset, the regeneration of bones is not guaranteed despite a GFD. Testing for CeD is recommended for all individuals with low BMD, recurrent fractures, and osteoporosis, even in the absence of G.I. symptoms.

## 7. Joints and Muscles

Joint manifestations have often been reported in patients with CeD. Symptoms are usually described in a general way as arthralgia rather than objective synovitis, although subclinical synovial effusion and sacroiliitis have also been reported [52] (Figure 2; Table 1). To date, the pathogenesis of joint complaints in CeD remains majorly speculative. Joint pain is a frequent musculoskeletal manifestation occurring in 20–30% of patients at CeD diagnosis. Studies investigating the role of GFD on joint pain are still conflicting. A Swedish survey on adults with CeD found that joint pain did not improve after initiating a GFD [53]. A study, conducted in the United States, reported response rates of 73% in children and 54% in adults for arthralgia and 75% in children and 69% in adults for arthritis, respectively [54]. The effect of a GFD in children was confirmed by the following two investigations. A total of 74 Italian children with CeD were examined for joint involvement in the knees, hips, and ankles [55]. Thirty-eight patients were on a gluten-containing diet (GCD) and thirty-six patients were on a GFD. Joint abnormalities were detected in 19 patients (50%) on a GCD and in 4 patients (11%) on a GFD. An Indian study evaluated early joint involvement in 60 children with newly diagnosed CeD and 60 children with CeD on a GFD for more than six months [56]. A total of 19 newly diagnosed patients (32%) had at least one joint abnormality at diagnosis, while only 2 patients on a GFD (3%) showed abnormalities.

Myalgia describes muscle aches and pain that can also compromise tendons, ligaments, and fascia. In untreated CeD, myalgia can be caused either by nutritional deficiencies or systemic inflammation. Subclinical enthesopathies might be frequent, and the entheseal site most frequently involved was the patellar (distal and proximal) [57]. Data on the frequency of myalgia in CeD and the effect of a GFD are widely missing. Only one study from the United States demonstrated that a strict GFD (≥24 months) achieved myalgia symptom resolution in all 157 investigated children with CeD, but only in half of 171 adults with CeD [54]. Overall, further research is necessary to clarify the association between myalgia and CeD.

Idiopathic inflammatory myopathies (IIMs) include a variety of acquired, chronic, and relapsing disorders characterized by skeletal muscle inflammation (myositis). Several studies reported an increased CeD prevalence in IIM patients. For example, Danielsson et al. evaluated the prevalence of CeD in IIM patients from Norway [58]. Four of the eighty-eight patients with IIMs (4.5%) had biopsy-confirmed CeD, which was significantly higher than the prevalence in the general population (0.53%) detected with a similar screening procedure. In the first systematic review of the literature presenting associations between CeD and IIMs, data for patients with IIMs and biopsy-verified CeD were explored for the effect of a GFD [59]. The results revealed that a GFD showed clinical improvement of IIMs in 14 of 24 patients (58%).

All in all, there is scientific evidence that untreated CeD is associated with joint pain and different muscle complaints. In the future, more investigations are needed to confirm these findings and to explore the pathogenesis of these complaints in CeD and the role of a GFD in resolving them.

## 8. Heart and Vessels

An increasing number of investigations have been published on cardiovascular involvement in CeD during recent decades (Figure 2; Table 1). Bernardi et al. focused their literature search on the pathophysiological bases of cardiovascular diseases (CVDs) [60]. The major evidence supports the theory of an increased cardiovascular risk in CeD due to the many mechanisms of myocardial injury, such as chronic malabsorption, abnormalities of intestinal permeability, and direct immune response against self-proteins. A systematic review presented in 2017 summarized results regarding the most frequent CVDs in untreated CeD [61]. The largest number of published documents concerned CeD in conjunction with cardiomyopathy (33 studies), thrombosis (27), cardiovascular risk (17), atherosclerosis (13), stroke (12), and ischemic heart disease (11). Most of them tended to resolve on a GFD, often in conjunction with the healing of small intestinal villous atrophy. However, in some cases, the alterations were irreversible, underscoring the need for CeD screening and treatment when cardiovascular issues of unknown etiology arise. In 2021, Fousekis et al. reported on studies related to thromboembolic complications and cardiovascular events associated with CeD [62]. The risk of cardiovascular diseases, stroke, myocardial infarction, and thromboembolism such as deep vein thrombosis and pulmonary embolism is higher in patients with CeD, while there is accumulating evidence that a GFD in CeD patients decreases the risk of these complications. Recent meta-analyses and original investigations revealed that CeD patients have an increased risk of overall CVD, including myocardial infarction and atrial fibrillation [63], although the exact link between CeD and stroke is scantly understood. Further studies are required in order to investigate the relationship between CeD and other cardiac arrhythmias such as ventricular arrhythmia. Furthermore, data about the association between CeD and cardiomyopathy, myopericarditis, and heart failure are still unclear.

Like adults, children with untreated CeD can be affected by cardiovascular abnormalities. For example, a retrospective evaluation of cardiac function was performed in 50 Indian pediatric patients at the diagnosis of CeD and after one year of GFD in comparison to 25 healthy controls [64]. On average, untreated CeD children had larger left ventricle diastolic dimension, reduced left ventricular ejection fraction, and a higher myocardial performance index compared to controls. After one year, GFD-compliant children showed improvement and non-compliant children showed persistence of cardiac dysfunction. Thirty Turkish children with CeD and thirty matched healthy children were enrolled to investigate the effect of CeD on myocardial functions and aortic elasticity parameters [65]. Isovolumetric relaxation time and isovolumetric contraction time ratios were significantly different between the groups. The myocardial performance index was also found to be statistically different.

Altogether, the risk of cardiovascular complications in both pediatric and adult patients with CeD appears to be higher compared to healthy controls, and a strict GFD may reduce the risk of CVD. In cases of CVDs of obscure etiology, clinicians’ awareness of possible CeD is warranted.

## 9. Kidney

CeD has been associated with a number of kidney diseases, such as IgA nephropathy, membranous nephropathy, diabetes nephropathy, and chronic kidney disease [66] (Figure 2; Table 1). A systematic review and meta-analysis showed that CeD patients have a higher risk of kidney diseases, especially diabetic nephropathy and IgA nephropathy [67]. In a long follow-up study from 1970 to 2015, Nurmi et al. investigated whether Finnish adults with biopsy-confirmed CD (n = 1072) were at higher risk of any kidney disease (glomerulonephritis, diabetic nephropathy, and interstitial nephropathy) than matched non-CeD controls (n = 3197) [68]. The prevalence of kidney disease in CeD subjects (3.5%) was significantly higher than that in controls (1.1%). Considering single disorders, significant differences in prevalence were found in glomerulonephritis (1.6% vs. 0.4%), IgA nephropathy (0.56% vs. 0.03%), diabetic nephropathy (1.8% vs. 0.6%), interstitial nephritis (0.47% vs. 0.09%), and end-stage renal disease (1.2% vs. 0.2%). Another study demonstrated that adult untreated CeD patients, symptomatic or not, have an increased risk of urolithiasis [69], likely due to hyperoxaluria [70]. An awareness of possible renal manifestations is recommended when treating patients with CeD.

In summary, the absolute risk of renal disease in CeD is low but significantly higher compared to control individuals. Screening for CeD in patients with kidney disease has not been recommended. The association between CeD and kidney disorders, the effect of a GFD, and pathogenesis need further investigation.

## 10. Nerves

Several neurological symptoms and alterations of the peripheral or central nervous system have been reported in untreated CeD patients, such as peripheral neuropathies, epilepsy, cerebellar ataxia, migraine, and cognitive impairment [71]. These conditions can be the only manifestations of CeD and are often under-recognized [72]. Updates on the relevant neurological manifestations of CeD have been presented by Patel et al. [73] and Gala et al. [74]. They are rare in children but as many as 36% of adult CeD patients present with neurological findings [75]. Although the exact pathogenetic link with CeD has not been clearly understood, there are several proposed mechanisms, such as the lack of essential nutrients, the production of antibodies against components of the nerves and brain, the opiate-like effects of gluten peptides in the brain’s immunochemical response due to inflammation, and effects on intestinal microbiota and their products.

Peripheral neuropathies and gluten ataxia are among the major neurological disorders mainly observed in adult CeD patients. A systematic literature review including 16 studies demonstrated that peripheral neuropathy was a manifestation of CeD in up to 39% of cases (13 studies), and gluten ataxia had a prevalence in CeD of up to 6% (9 studies) [76]. Adherence to a GFD appeared to improve the symptoms of both disorders. Epilepsy has also been associated with CeD, with the prevalence of CeD ranging from 1% to 8%. An epidemiological study, based on the Swedish register of CeD, showed that, when epilepsy was restricted to those with both a diagnosis of epilepsy and an independent record of antiepileptic drug prescriptions, CeD was associated with a 1.43-fold increased risk of epilepsy [77]. Data from a systematic review revealed that epilepsy was 1.8 times more frequent in CeD patients and, vice versa, CeD was over 2 times more prevalent in patients with epilepsy than controls [78]. Therefore, serological screening for CeD is suggested in patients with epilepsy of unknown etiology, since these patients may benefit from a GFD. Not only adults but also children and youths with CeD can be affected by epilepsy. For example, an Italian population-based study, including 1215 young CeD patients and 6075 matched reference individuals, showed a 2.6% prevalence of epilepsy in CeD patients and 1.3% in controls [79]. Thus, young patients with epilepsy without a clear etiology should be screened for CeD.

An association was also found between CeD and migraines and headaches, which usually occur periodically and come on quickly. A systematic review by Zis et al. reported that 26% of CeD adults and 18% of CeD children suffer from headaches [80]. A GFD seems to be effective in promoting the resolution of headaches in up to 75% of patients. In an Iranian study, 1000 adult patients with biopsy-verified CeD and 1000 matched non-CeD individuals were investigated for the prevalence of migraine and headaches [81]. The prevalence of migraine in CeD patients was significantly higher than in controls (20.7% vs. 11.9%) and more prevalent in females with CeD compared to males with CeD (80% vs. 19%). Headache was more common in CeD than in controls (34% vs. 27%) and more prevalent in females than in males (72% vs. 28%).

Little information is available about the association of CeD with other neurological conditions, such as transient cognitive impairments (“brain fog”) of memory, attention executive function, and the speed of cognitive processing. Nutritional deficiencies, increases in inflammatory cytokine levels, and low brain serotonin levels are among the proposed pathogenetic mechanisms [82]. Recently, a nationwide study from the United States examined 1143 adult CeD patients on a GFD for neurocognitive impairment after gluten ingestion [83]. All in all, 89% of patients reported having neurocognitive symptoms. The most common word descriptors were difficulty concentrating, forgetfulness, and grogginess. The associations of CeD with tremor and restless leg syndrome are scarcely described [84], and their prevalence and clinical correlates have not been established. These complaints need further examination.

In summary, most studies confirmed a higher prevalence of peripheral neuropathies, gluten ataxia, epilepsy, migraine/headaches, and cognitive impairments in patients with CeD. A strict GFD is an effective first-line treatment and only very few patients will require additional treatment. The consulting neurologist should have up-to-date knowledge of the association between CeD and unexplained neurological diseases and can play an important role in the identification of patients with undiagnosed CeD.

## 11. Psyche

Numerous studies have identified associations between CeD and psychiatric disorders in both children and adults (Figure 2; Table 1). Although the pathogenesis of psychiatric disorders in CeD is not completely understood, it has been hypothesized that the immune reaction might produce inflammation and damage in the brain, promoting the dysfunction of the gut–brain axis. However, the precise mechanisms underlying these reactions are not known. A systematic review and meta-analysis, including 37 scientific articles, presented the prevalence data of psychiatric manifestations of CeD [85]. Compared to healthy controls, individuals with CeD showed increased risks for autistic spectrum disorder, attention deficit hyperactivity disorder, depression, anxiety, and eating disorders. A nationwide cohort study in Sweden of 10,903 children demonstrated that patients with CeD had a 1.4-fold greater risk of future psychiatric disorders [86]. Childhood CeD was identified as a risk factor for mood disorders, anxiety disorders, eating disorders, behavior disorders, autistic spectrum disorder, attention deficit hyperactivity disorder, and intellectual disability. A large multi-center database from 26 major integrated healthcare systems, including 360 hospitals in the United States, was used to describe the epidemiology of common psychiatric disorders in CeD [87]. Of the 37,465,810 patients in the database between 2016 and 2020, there were 112,340 (0.30%) individuals with CeD. When compared with patients with no history of CeD, patients with CeD were more likely to have a history of anxiety, depression, bipolar disorder, attention deficit hyperactivity disorder, eating disorder, and childhood autistic disorder.

Schizophrenia is one of the psychiatric disorders with the most robust relationship to CeD (the so-called bread madness). Since 1953, there have been several case reports of CeD patients who significantly recovered from schizophrenia with the implementation of a GFD. A systematic review and meta-analysis by Wijarnpreecha et al. reported an overall significantly increased risk of schizophrenia in CeD patients [88]. Conversely, the evaluation of more than 10,000 patients with schizophrenia revealed an increased risk for CeD in these patients [89]. Anxiety and depression also are common psychological complaints in patients with untreated CeD. A study from the United States utilized the Revised Children’s Anxiety and Depression Scale (RCADS) to investigate anxiety and depression symptom rates in 175 children with biopsy-confirmed CeD [90]. Self-reported RCADS scores showed 39% of children had clinically significant concerns for anxiety or depression.

Profound and debilitating fatigue is one of the most common extra-GI manifestations reported among individuals with CeD. A recent review revealed that fatigue was significantly greater in patients with CeD compared to healthy controls with a prevalence ranging from 8 to 100% [91]. A GFD seems to reduce fatigue, but the existing data are limited. Several studies demonstrated the increased prevalence of eating disorders in CeD patients. A systematic review and meta-analysis (23 observational studies) of the prevalence of eating disorders in patients with CeD indicated that the pooled prevalence of eating disorders and bulimia nervosa in patients with CeD was 8.9% and 7.3%, respectively [92]. Moreover, the risk of anorexia nervosa in patients with CeD, and, vice versa, the risk of CeD in patients with anorexia nervosa, was significantly higher than in the healthy population.

Several studies on children have demonstrated the association between CeD and behavioral problems, such as autistic-like behavior. These patients appear unhappy and introverted, with difficulties in socializing and communicating with others. These symptoms can improve with a GFD. Several clinical studies investigated the association between autism spectrum disorder (ASD) and CeD in both children and adults. A systematic review, including 17 articles that evaluated patients with ASD or patients with CeD, has been presented by Quan et al. [93]. Overall, most studies had small sample sizes and reported no evidence for an association between the two conditions. However, a limited number of population-based studies of higher quality suggested a potential association between CeD and ASD. Large-scale investigations are needed to confirm the link between CeD and ASD and the benefits of a GFD.

There is little knowledge about the association between CeD and attention-deficit/hyperactivity disorder (ADHD). Kumperscak et al. [94] examined 102 children and adolescents with ADHD for CeD demonstrating that the prevalence of CeD in these patients was similar to that of the general population. However, children with ADHD showed high levels of anti-gliadin antibodies, suggesting that gluten may play a role in ADHD. Kristensen et al. examined 26 CeD patients before starting a GFD and after at least 12 months of a GFD, using a specific ADHD questionnaire [95]. They revealed that before a GFD, CeD patients had significantly higher ADHD scores than healthy controls. After a GFD, the scores of CeD patients improved and were not significantly different from the controls.

In conclusion, CeD patients present signs of schizophrenia, anxiety, fatigue, and eating disorders more frequently compared to healthy individuals and may benefit from a GFD. The situation regarding ASD and ADHD remains unclear and needs further investigation. Moreover, further research is necessary to understand the pathophysiology of psychiatric manifestations in CeD.

## 12. Fertility and Pregnancy

Studies regarding the infertility of men with CeD are scarce and contradictory, and further investigations are needed to obtain clear results. The exact risk estimate of infertility in women with CeD and the role of a GFD remains ambiguous [96] (Figure 2; Table 1). The weaknesses of the results have been the variable definitions of infertility, the small sizes of study groups, and a lack of control groups. Moreover, the diagnosis of CeD often includes only serological testing and not duodenal biopsy. A meta-analysis by Singh et al., including nine studies published until 2014, showed that the prevalence of CeD in women with overall infertility was 2.3% and that in women with unexplained fertility was 3.2% [97]. Women with overall infertility had 3.5 times higher odds and those with unexplained infertility had 6 times higher odds of having CeD in comparison with the control population. In a later systematic review and meta-analysis, Castano et al. analyzed data from 23 scientific articles published until 2019 [98]. The results demonstrated that the prevalence of a positive CeD-specific serology was similar for overall infertility and unexplained infertility with a pooled proportion of 1.3–1.6%. This implied three-times-higher odds of having CeD in women with infertility when compared to the controls. The authors of both meta-analyses concluded that these findings support a higher risk of CeD in infertile women, particularly when they have unexplained infertility.

However, a recent systematic review with a meta-analysis of the prevalence of CeD in women with infertility revealed that the pooled prevalence of biopsy-confirmed CeD was 0.7% in women with overall infertility [96]. Among women with unexplained infertility, the pooled prevalence of biopsy-confirmed CeD was 0.6%. After including studies where CeD had been defined only by serology, the pooled prevalence of CeD was 1.1% in women with any infertility. In conclusion, these findings suggested that CeD was not more common in infertile women than in the general population.

The effect of a GFD has been differently judged. A retrospective study, performed in a Spanish infertility clinic, explored the effect of a GFD in women with CeD who experienced recurrent implantation failure [99]. The in vitro fertilization data of 19 women following a GCD were compared with those of 10 women on a GFD. The results revealed significant differences between the GCD and GFD in terms of the live birth rate (0% vs. 60%). In contrast, a study from the United States on 28 women with seropositive CeD who underwent in vitro fertilization demonstrated that those on a GFD (n = 3) were not different in outcomes; fertilization rates (82% vs. 84%) were similar in both groups [100].

A growing number of studies have investigated the association between CeD and adverse pregnancy outcomes. Nutritional deficiencies and anemia, often occurring in active CeD, as well as the impairment of physiological processes during the implantation of an embryo and/or during the development of the placenta, have been considered the main causes of pregnancy complications [101,102]. A recent meta-analysis, including 14 cohort and 4 case–control studies, revealed that the relative risks of stillbirth, spontaneous abortion, fetal growth restriction, preterm delivery, and lower birth weight were significantly higher in pregnant women with CeD compared to non-CeD women [103]. Only undiagnosed CeD raised these risks, according to the subgroup study, while early CeD diagnosis was not associated with any worse pregnancy outcome. The following investigations exemplarily demonstrate the relationship between pregnancy problems and CeD.

A nationwide Danish retrospective study, including 6319 women diagnosed with CeD and 63,166 matched controls, was performed to assess the rate of stillbirth before and after the diagnosis of CeD [104]. Before CeD diagnosis, women had an excess rate of stillbirth compared to non-CeD women (0.45% vs. 0.29%). After CeD diagnosis, no significant difference in the frequency of stillbirth was found (0.32% vs. 0.29%). These results suggest that early diagnosis and treatment of CeD is important for preventing stillbirth. A retrospective cohort study from the United States aimed to compare the rates of spontaneous abortions (S.As.) in women with CeD and controls [105]. A total of 245 women with CeD and 488 women with no history of CeD were asked to complete an anonymous online survey on the frequency of S.As. The rate was 50.6% in the CeD group, which was significantly higher than the rate of 40.6% in the control group. Of the 124 women in the CeD group who had S.As., 105 (85%) reported that it was before their diagnosis of CeD. The group of CeD women with early pregnancy loss had a more frequent allelic 4G variant of PAI-1 and a more frequent mutant genotype [106].

The prevalence of fetal growth restriction (FRG) was assessed in a Slovenian retrospective study, including 120 women with biopsy-confirmed CeD and 59 matched control women without any gastrointestinal disorder [107]. All participants were asked to complete an anonymous questionnaire specifically developed for the study. The results indicated that FGR occurred significantly more often in women with CeD (11.7%) compared to the healthy controls (1.7%). A retrospective study from Israel evaluated the frequency of preterm delivery (PTD) and low birth weight (LBW) in CeD and healthy controls [108]. Among 243,682 deliveries which met the inclusion criteria, 212 neonates (0.08%) were born to mothers with known CeD. The results revealed that maternal CeD was an independent risk factor for PTD and LBW: women with CeD had significantly higher rates of PTD (10.4%) and LBW (14.2%) compared to the control group (6.9% and 6.7%, respectively). Several other studies also showed a higher frequency of PTD and LBW in the offspring of CeD women, but the differences with controls were not always significant. A couple of investigations examined whether relations between CeD and the rate of cesarean delivery exist. The great majority of studies found that both issues are not associated, but further research is necessary to confirm this finding.

In conclusion, most of the studies showed a higher prevalence of stillbirth, S.A., FRG, PTD, and LBW in women with unrecognized CeD. Therefore, these conditions can be regarded as a “risk indicator” for CeD, and physicians should start screening for it, even when G.I. symptoms are not present. An early diagnosis of CeD minimizes the possibility of unfavorable pregnancy outcomes among women of childbearing age via the adoption of a GFD.

## 13. Discussion

Originally, CeD was considered a rare disorder of the small bowel affecting only children and characterized by chronic diarrhea and malabsorption of nutrients. Today, it is recognized as a disease that can occur both in children and adults, at any age. Step by step, starting with dermatitis herpetiformis and bread madness (schizophrenia), the association of CeD with extra-GI manifestations has been realized including a wide clinical spectrum that involves almost the whole body. While most classical G.I. manifestations are frequent signs of CeD in both children and adults, a couple of extra-GI manifestations are presented more often in adults compared to children. Some extra-GI manifestations such as dental enamel defects, osteoporosis, and fatigue are reported quite often, and others such as hearing loss, ocular manifestations, myocardial infarction, and kidney disorders are rare. Some patients have only one or two symptoms, and others have many symptoms simultaneously. In some cases, extra-GI symptoms are the only clinical pictures of CeD. Patients without G.I. complaints are at high risk of significantly delayed diagnosis or they even remain undiagnosed. Moreover, non-adherence to a strict GFD additionally contributes to the appearance of these manifestations in already diagnosed CeD patients. Several international studies have reported that the rate of non-compliance with a GFD ranged between 10% and 60% [109]. On the other hand, a GFD ameliorates most extra-GI symptoms of adult CeD patients [110].

An increased awareness of the variety of extra-GI manifestations among medical practitioners is essential to enhance the rate of CeD diagnosis. Medical specialists such as dermatologists, dentists, otorhinolaryngologists, orthopedists, gynecologists, neurologists, and psychiatrists should know the possible CeD-associated disorders and could play an important role in identifying undiagnosed patients. Because around half of patients with CeD are undiagnosed and untreated, CeD has been suggested as appropriate for mass screening. Accordingly, all individuals regardless of symptoms should undergo serological screening for CeD, and those who subsequently test positive should undergo endoscopy with duodenal biopsy. However, the role of widespread population screening remains controversial [111]. The arguments against mass screening are unresolved questions such as the appropriate age and the interval of screening, relatively high false-positive rates due to the specificity of serological tests <100%, and finally high costs. An alternative to population screening is an active case-finding approach: screening has been recommended for persons when their close relatives have a confirmed diagnosis of CeD, and for persons with autoimmune diseases associated with CeD [112]. Some extra-GI manifestations such as dental enamel defects, psoriasis, osteoporosis, epilepsy, schizophrenia, and recurrent pregnancy problems should be included in the active case-finding approach.

The precise pathophysiological mechanisms underlying the comorbidities of CeD are only partly known. Some extra-GI manifestations are direct consequences of the malabsorption of essential nutrients, especially minerals and vitamins. Examples include dental abnormalities, osteoporosis, and iron anemia. The pathophysiological mechanisms of other manifestations such as infertility and neuropsychiatric disorders are poorly established. Their etiology might be complex and seems to be more than a direct consequence of malabsorption. CeD-mediated immune responses have been proposed to be indirectly related to extra-GI manifestations, e.g., by the release of cytokines and chemokines into the blood stream via adaptive and innate immunity. Moreover, interference in brain processes, dysfunction in the gut–brain axis, and involvement of the endogenous opiate system are in discussion, but the precise mechanisms underlying these reactions are still not known. Another unresolved question is the contribution of genetic and environmental factors.

Most CeD-mediated symptoms can be alleviated by a GFD, often in conjunction with the healing of the small intestinal mucosa [113]. G.I. symptoms show higher rates of improvement compared to extra-GI symptoms. However, some of the CeD-associated disorders are irreversible unless CeD is treated in time. For example, dental enamel defects in children and adults and osteoporosis in adults will remain permanent if not treated early. Therefore, early recognition of CeD and close attention to GFD adherence are important for symptom resolution and the prevention of severe complications. However, a minority of cases do not reveal improvement despite a GFD. Reasons might be non-strict adherence to a GFD, additional causes for the manifestations or the presence of refractory CeD.

All in all, many issues concerning the various clinical features of untreated CeD are still unresolved and need to be studied by future research. Lack of knowledge has been specifically observed in extra-GI manifestations concerning their prevalence rates, pathogenic mechanisms, genetic and environmental factors, and the effects of a GFD.

## Figures and Tables

**Table 1:** Extra-gastrointestinal manifestations of GFD and the possible effect of GFD.

	Extra-Gastrointestinal Manifestation	Gluten-Free Diet Effect
Oral cavity	delayed dental eruption dental enamel defects (DEDs), dental caries, dental plaque, and periodontitis recurrent aphthous stomatitis (RAS) angular cheilitis, atrophic glossitis burning tongue, xerostomia, mucosal lesions lymphocytic sialadenitis	Early diagnosis of CeD and treatment with a GFD might prevent the development of DEDs
Nose and ears	sensorineural hearing loss obstructive sleep apnea nasal septal perforation and epistaxis	Symptom resolution, except for sensorineural hearing loss
Eyes	nyctalopia dry eye, cataract thyroid-associated orbitopathy uveitis central retinal vein occlusion neuro-ophthalmic manifestations	Unclear
Skin and Hair	dermatitis herpetiformis (DH) chronic urticaria atopic dermatitis psoriasis rosacea alopecia areata	Improvement
Bones	bone pain and fracture osteopenia and osteoporosis	Recovery of bone density if GFD is timely
Joints and Muscles	arthralgia and joint pain idiopathic inflammatory myopathies (IIMs)	Unclear
Heart and Vessels	cardiomyopathies atherosclerosis stroke and ischemic heart disease deep vein thrombosis and pulmonary embolism	A GFD in CeD patients decreases the risk
Kidney	membranous nephropathy IgA nephropathy diabetes nephropathy chronic kidney disease urolithiasis	Untreated CeD patients have an increased risk of urolithiasis, likely due to hyperoxaluria
Nerves	peripheral neuropathies cerebellar ataxia epilepsy migraine cognitive impairment	Adherence to a GFD appeared to improve symptoms
Psyche	autistic spectrum disorder (unclear) attention deficit hyperactivity disorder (unclear) depression, anxiety, fatigue eating disorders schizophrenia	Schizophrenia, anxiety, fatigue, and eating disorders may benefit from a GFD
Fertility and Pregnancy	female infertility stillbirth, spontaneous abortions (S.As.) fetal growth restriction (FRG), preterm delivery (PTD), low birth weight (LBW)	GFD minimizes the risk of adverse pregnancy outcomes

## Data Availability

The original contributions presented in the study are included in the article; further inquiries can be directed to the corresponding author.
